# Proton Low Field NMR Relaxation Time Domain Sensor for Monitoring of Oxidation Stability of PUFA-Rich Oils and Emulsion Products

**DOI:** 10.3390/foods10061385

**Published:** 2021-06-15

**Authors:** Maysa T. Resende, Tatiana Osheter, Charles Linder, Zeev Wiesman

**Affiliations:** Phyto-Lipid Biotechnology Lab (PLBL), Department of Biotechnology, Faculty of Engineering Sciences, Ben Gurion University of the Negev, Beer Sheva 84105, Israel; mteixeiraresende@gmail.com (M.T.R.); osheter@post.bgu.ac.il (T.O.); charles.linder@gmail.com (C.L.)

**Keywords:** emulsions, PUFA, ^1^H LF NMR, time domain, oxidation, monitoring, chemistry and morphology arrangement

## Abstract

The nutritional characteristics of fatty acid (FA) containing foods are strongly dependent on the FA’s chemical/morphological arrangements. Paradoxically the nutritional, health enhancing FA polyunsaturated fatty acids (PUFAs) are highly susceptible to oxidation into harmful toxic side products during food preparation and storage. Current analytical technologies are not effective in the facile characterization of both the morphological and chemical structures of PUFA domains within materials for monitoring the parameters affecting their oxidation and antioxidant efficacy. The present paper is a review of our work on the development and application of a proton low field NMR relaxation sensor (^1^H LF NMR) and signal to time domain (TD) spectra reconstruction for chemical and morphological characterization of PUFA-rich oils and their oil in water emulsions, for assessing their degree and susceptibility to oxidation and the efficacy of antioxidants. The NMR signals are energy relaxation signals generated by spin–lattice interactions (T_1_) and spin–spin interactions (T_2_). These signals are reconstructed into 1D (T_1_ or T_2_) and 2D graphics (T_1_ vs. T_2_) by an optimal primal-dual interior method using a convex objectives (PDCO) solver. This is a direct measurement on non-modified samples where the individual graph peaks correlate to structural domains within the bulk oil or its emulsions. The emulsions of this review include relatively complex PUFA-rich oleosome-oil bodies based on the aqueous extraction from linseed seeds with and without encapsulation of externally added oils such as fish oil. Potential applications are shown in identifying optimal health enhancing PUFA-rich food formulations with maximal stability against oxidation and the potential for on-line quality control during preparation and storage.

## 1. Introduction

Polyunsaturated fatty acids (PUFAs) such as α-linolenic acid (ALA), eicosapentaenoic acid (EPA) and docosahexaenoic acid (DHA) are important nutrients that regulate a wide variety of biological functions, from blood pressure to the development and functioning of the brain and nervous system [1,2,3,4]. Paradoxically, although PUFAs are very important for health, they also undergo oxidation during storage to form toxic oxidant products [5]. Our major research objective has been to develop a facile and efficient analytical tool to measure directly on non-modified highly nutritious PUFA containing food samples, their chemical and morphological arrangements using energy relaxation times controlled by spin–spin and spin–lattice relaxation on a single graph, to guide the controlling of nutritional stability and prevention of oxidation during preparation, shelf life and digestion. This is related to the functionality and oxidant stability of PUFA foods being based on their food components internal physical and morphological arrangements [6,7].

In this regard we have recently shown the efficacy of ^1^H LF NMR energy relaxation sensing and signal reconstruction algorithms for generating the complex chemical and morphological arrangement of the polysaccharides, proteins, sugars and fats of biomass used for biofuels on a single 2D energy relaxation graph [8,9]. In the present paper we review our work using ^1^H LF NMR energy relaxation technology with modified signal reconstruction algorithms for characterizing PUFA and their triglyceride oils and oil in water (O/W) emulsions with respect to their internal chemical and morphological arrangement. This correlates to their susceptibility to oxidation and antioxidant efficacy in food preparation and storage.

### 1.1. ^1^H LF NMR Relaxation Sensor Technology

^1^H ^1^H LF NMR is a rapid, non-destructive technology extensively used in the food, polymer, petroleum and pharmaceutical industries [10,11]. It is widely used in quality control for the determination of solid-to-liquid and oil-to-water ratios in materials as diverse as oil-bearing rock, food emulsions and plant seeds [12].

The field of ^1^H LF NMR relaxometry is a powerful tool for identifying molecular species and to study their dynamics even in complex materials. This relates to the measurement of energy relaxation constants as a consequence of interactions among nuclear spins and between them and their surroundings matrix. Longitudinal magnetization returns to equilibrium following application of a radio frequency field because of energy transferred to the lattice, and transverse energy relaxation arises from spin–spin interactions following a 90° pulse. The time constants for longitudinal and transverse energy relaxations are T_1_ and T_2_ respectively.

Relaxation time distribution experiments range from simple and rapid one dimensional (1D) tests to more complicated multidimensional ones. One-dimensional tests use constant intervals between pulses, allowing for either longitudinal or transverse relaxation to be evaluated, whereas in multidimensional experiments, the signal is measured as a function of two or more independent variables, allowing the spin system to evolve under different relaxation mechanisms [13]. In biological samples, spins exist in a variety of different environments, giving rise to a distribution of relaxation times in which the measured relaxation decay is a sum of contributions from all spins [14].

Most commonly applied, 1D NMR tools are based on either acquisition of the free induction decay signal following a 90° pulse, or pulse sequences such as the spin echo [15], CPMG [16,17] or inversion/saturation recovery [18].

More recently, a new two-dimensional (2D) energy relaxation time distribution pulse sequences has been suggested, including T_1_ and T_2_ [13], T_2_-store-T_2_ [19] and T_2_-Diffusion measurements [20].

**Figure 1:**
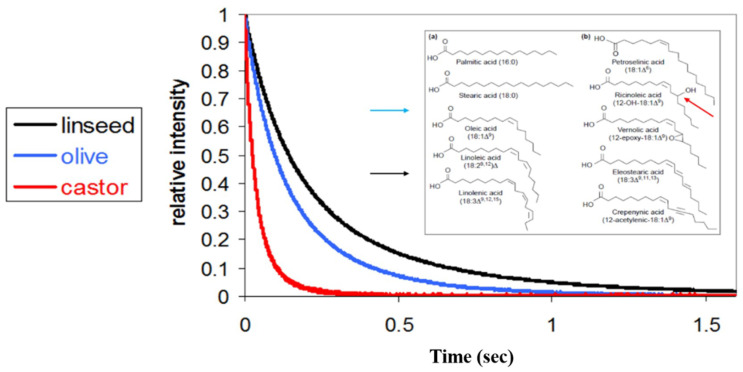
Effect of dominating oil’s fatty acids on ^1^H LF NMR relaxation curve.

The basic principle of the ^1^H LF NMR relaxation TD sensor, is that the oils FA’s composition directly affects and dominates the proton energy relaxation time. This principle is shown by three different oils in Figure 1. For the most viscous castor oil composed of about 90% ricinoleic acid (12-OH-18:1) with hydroxyl groups, has T_2_ aliphatic chain proton energy relaxation time values significantly shorter than the other two oils. Monounsaturated olive oil, of lower viscosity, with about 60% oleic acid (18:1) shows an intermediate T_2_ relaxation time, while PUFA-rich linseed oil of lowest viscosity, with about 55% linolenic acid (18:3) is the most mobile and the T_2_ relaxation time is the longest of the three oils. This clearly shows the effect of both the chemical composition and its structural arrangement on the NMR signal generation in terms of proton time spin–spin energy relaxation values. However, significantly more information on the oil’s chemical and morphological material arrangements can be obtained from this data, as described below, by more advanced algorithms for signals collection and T_1_ and T_2_ spectrum reconstructing. It is clear that further enhanced data processing of the progress and changing relaxation times along the curves in Figure 1, contains significant additional material information about the oils.

New computing approach and algorithms for efficient and better processing of ^1^H LF NMR signals collected energy relaxation data were developed in our research team. This new computing approach applies combined L_1_-norm and L_2_-norm regularization to find sparse solutions, using a formulation suitable for the PDCO solver (primal-dual interior method for convex objectives). Using these algorithms, we were able to better solve inverse Laplace transformation problem and to produce and generate 1D and 2D T_1_ vs. T_2_ graphical representation of the proton mobility in simple liquid and solid and complex samples as well. These proton relaxation data processing methods significantly improved the common L_2_-norm (least squares) results and enable to separate many additional new time domains (TD) in the tested samples.

^1^H LF-NMR spectroscopy generates energy relaxation time exponential decay signals, from which the PDCO solver transforms the exponential decay curves components into spectra of material samples that can distinguish between different chemical and morphological components of the analyzed sample. Transforming relaxation curves into 1D spectra of T_1_, T_2_ and 2D T_1_ vs. T_2_ spectra is an ill posed problem of the inverse Laplace transform (ILT) [21].

**Figure 2:**
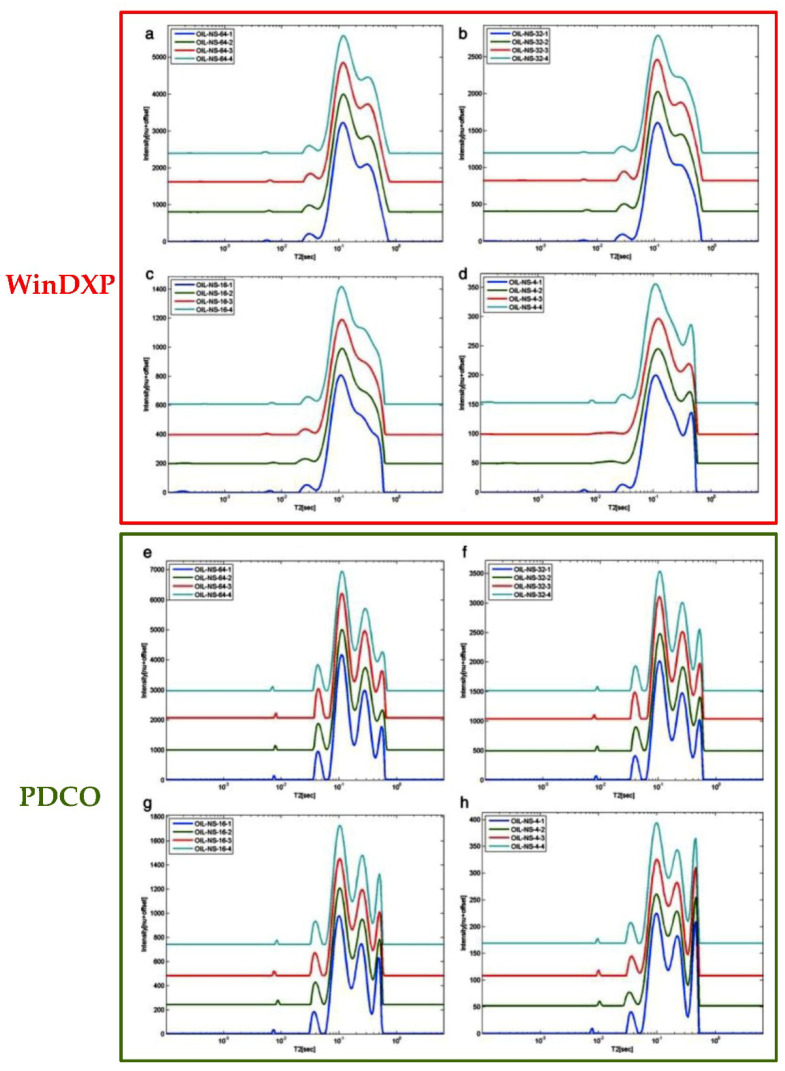
Comparison of 1D WinDXP T_2_ spectra (**a**–**d**) (RED) vs. PDCO T_2_ (**e**–**h**) (GREEN) data processing of rapeseed oil. Reprinted with permission from ref. [21]. Copyright 2013 Wiley.

This inverse problem, like all inverse problems, is complex to solve for at least two major reasons: (1) any set of measurements may be consistent with different relaxation times, i.e., the solution of the inverse problem at hand is not unique; (2) finding a solution may require exploration of a huge parameter space. These issues must be resolved with an appropriate solver, which until recently the most common numerical method implemented is based on L_2_-norm regularization [12,21]. Sparse representation methods, however using L_1_ regularization and convex optimization are a relatively new approach for effective analysis and processing of digital images and signals. A numerical optimization method for analyzing LF NMR data by including non-negativity constraints and L_1_ regularization and by applying a convex optimization solver PDCO, which allows general linear constraints to be treated as linear operators, was shown to provide better resolved and more accurate solutions when compared to existing methodologies [21]. An example of the latest methodologies analysis of system relaxation data acquired using LF NMR is presented in Figure 2a–h for the common system based on WinDXP compared to the novel PDCO solver, respectively. A rapeseed oil sample was chosen as the chemical model for comparison. The solutions are ordered in each graph by descending signal to noise ratio (SNR) values. In contrast to WinDXP solution that produced only two main moderately resolved peaks, the PDCO solutions have four distinct resolved peaks [21]. As can be seen, all four repetitions of the PDCO solutions are highly repeatable and stable. PDCO formulation provides better resolved relaxation time distributions and more accurate solutions. Furthermore, the accuracy of the PDCO solver spectral generation was demonstrated in both 1D and 2D T_1_-T_2_ simulation studies [22,23].

**Figure 3:**
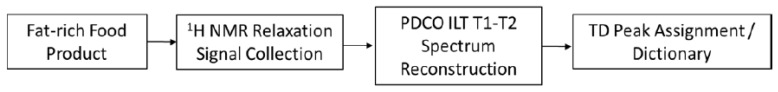
Concept and main components of ^1^H LF NMR sensor for food application.

In brief, the present developed ^1^H LF NMR energy relaxation TD sensor system consists of different interconnected components as shown in Figure 3. In effect: A fat-rich food product is initially used for T_1_ and T_2_ proton energy relaxation signals collection from exponential decay curves generated by the LF NMR unit [21,24]. Then the exponential decay data of ^1^H relaxation signals is processed by an inverse Laplace transformation using a PDCO solver with optimized regularization parameters for reconstruction of 1D and 2D T_1_-T_2_ spectra on a single graph [22,23]. The 2D T_1_-T_2_ graph is presented with numerical values for each separate TD peak. Following this data processing and reconstruction, each TD peak assignment is conducted according to referenced previous work, for the different segmental motions of the FA for pure FAs and oils described in Figure 1 and Figure 2, and their respective time relaxation values. This was further supported by HF ^1^H NMR in an intensive study for assignment of T_1_ and T_2_ values of common SAFA, MUFA and PUFA of various chain length [25]. Peak assignment for O/W emulsion will be described and demonstrated in subsequent sections.

### 1.2. Determination of Chemical and Physical Structure of Oils and Emulsions by the ^1^H LF NMR TD Sensor

**Figure 4:**
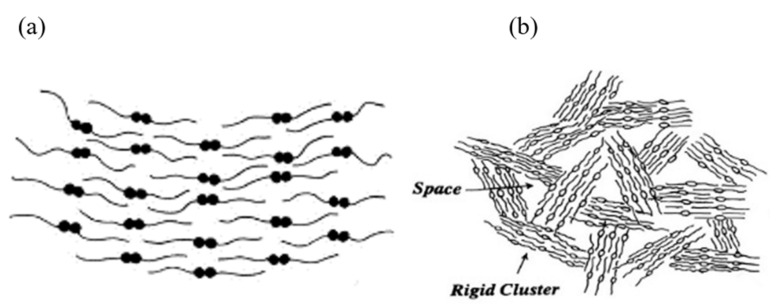
(**a**) α Linolenic acid dimers aggregate head-to-head to tail-to-tail quasi smectic liquid crystal and (**b**) microstructural morphology [25,26].

The ^1^H LF NMR technology described in this review is related to the need to monitor the internal physical/morphological arrangements of biological and nutritional oils as it strongly influences their chemical, oxidant and physical properties [26]. The different molecular structures monitored are the arrangements of the amphiphilic chains of the oil’s FAs whether saturated (SFA), MUFA or PUFA, and also their liquid bulk oil phase aggregate structures. These structures are formed by the carboxylic group’s head groups that hydrogen-bond to form a dimer of two FAs in a hydrophobic environment of the alkyl chains [26]. The head-to-head dimers aggregate as quasiliquid crystal clusters alternating head to tail arrangement forming aggregates that determine the FAs (Figure 4a), which further aggregated with void spaces to form a microstructural morphology resembling a house of cards (Figure 4b) [26].

**Figure 5:**
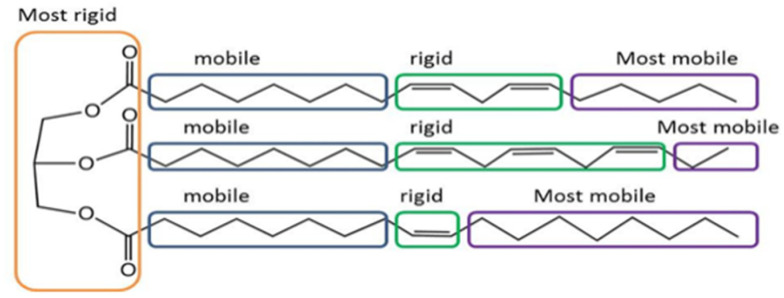
PUFA-rich TAG structure and segmental rigidity mobility [27].

The ^1^H LF NMR as applied to highly complex PUFA structures, for example, can detect their four different rigidity-mobility segments along the alkyl chain [25] where the most physically rigid microstructures are the head groups and secondly the alkyl chains double bonds. The more mobile segments are the saturated aliphatic chains between the head segment and the double bonds with the aliphatic tail chain, from the outer most double bond, is the most mobile segment [25,26]. This is also applicable to food oils composed of triacylglycerol (TAG) molecules that have structures characterized by a glycerol molecule that bridges three FAs chains. A PUFA-rich TAG structure (Figure 5) consists of four different rigidity-mobility segments with the glycerol segment as the most rigid microstructure with the double bonds the second most rigid [27]. The aliphatic chain between the glycerol and double bonds is more mobile as described above for PUFA (Figure 4), without glycerol wherein the aliphatic tail chain segment is the most mobile.

**Figure 6:**
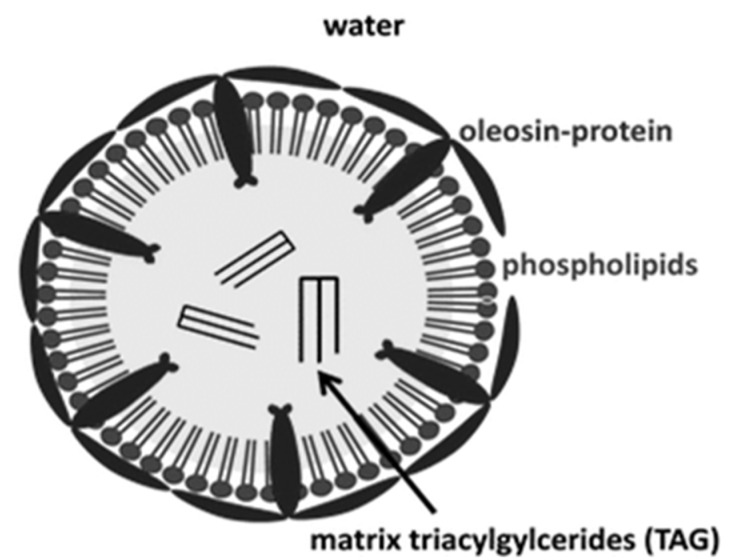
Schematic demonstration of an oleosome vesicle structure [33].

A common morphological form of PUFAs are oil-in-water (O/W) and water-in-oil (W/O) emulsion structures. Emulsions are essential components in a vast number of important commercial products such as foods, cosmetics, pharmaceuticals and petroleum [28,29,30]. Monitoring their chemical morphological structure by LF H1 NMR as described in this review has many applications in the preparation of optimal PUFA emulsion products. Emulsions can be defined as a mixture of two or more immiscible liquids in which the minor component is dispersed as small droplets throughout the other [31]. The O/W emulsion consists of oil droplets dispersed in an aqueous phase as in common commercial O/W emulsions such as mayonnaise, milk, ice-cream and sauces. The W/O emulsion is defined by water droplets dispersed in an oil phase as in butter and margarine [32]. O/W Emulsions may also be prepared with complex aggregate structures of oleosomes derived from seed oleosomes, found in seed oil bodies such as linseed, which are rich in the PUFA α-linolenic acid (ALA). In Figure 6 a schematic figure of O/W emulsion oleosome vesicle with an outer surface layer of an amphiphilic oleosin protein associated with water from outside and polar heads of phospholipids coating a core matrix of hydrophobic TAGs [33]. 

### 1.3. Oxidation of PUFA-Rich Oils and O/W Emulsions Food Products

In the present review paper, we show how the complex arrangement of the different phases and components of emulsions can indeed be further characterized using a novel ^1^H LF NMR energy relaxation time domain (TD) sensor including an optimized reconstruction program for generating chemical morphological fingerprinting maps. In this section we describe the complex oxidation mechanisms of PUFA rich products that are monitored by ^1^H LF NMR technology of this review: For example, in highly complex O/W emulsions of oil bodies extracted from linseed [24,27,34]. PUFA-rich oils found in many commercial products are highly susceptible to oxidation via complex free radical chain reactions characterized by an initiation, a propagation sequence and termination steps [24,27]. The initiation of oxidation may be catalyzed/triggered by relatively high temperatures, light exposure, transition metals and other initiators. Initiation begins on a PUFA molecule’s allylic carbons by hydrogen radical (H˙) abstraction, forming an akyl radicals (R˙), resulting in conjugations of PUFA’s double bond’s. The hydrogen atom attached to bisallylic carbon on PUFA requires the least amount of energy to be removed and is the molecular site of oxidation initiation [35,36]. The propagation step then includes the reaction between oxygen molecules and the akyl radicals, creating a peroxy radical (ROO˙). This radical can later react with another hydrogen atom from a different FA chain, thus propagating the reaction onwards. The oxidation products of the subsequent propagation and termination steps are responsible for the off-flavor in oxidized PUFA-rich products and are also associated in biological systems/tissues with pathological processes such as sclerosis, degradation of biologically-active proteins and the aging processes [37]. During the termination step of the free radical chain reaction, carbon radicals react with each other forming a covalent bond to produce stable species.

The susceptibility of PUFA oxidation in many food products is well known and there is an effort to reduce it by the localization of reactants inside the food matrix, one major approach is by O/W emulsions [38]. Oil in water emulsions are widely found in foods and supplements and in many cases show improved stability against oxidation as compared to the pure oils [35]. In most reports emphasis is given to the interfacial region, which is the contact between the oil phase and the aqueous phase, as a critical area of the system regarding lipid oxidation. This is due to the observation that oil droplet size (and therefore interfacial area) or emulsifier type and interfacial surfactant composition including protein-stabilized interfacial layers, significantly affect an emulsion’s lipid oxidation [38]. There have been many studies on the effect of emulsifiers on oxidant stability of O/W emulsion and the functionality of the interfacial region structure to control oxidation, and how this affects the general mechanisms of lipid oxidation. Yet, many apparent contradictions on the interfaces functionality have been published, which can be partially attributed to the structural complexity of emulsion systems and to its complex behavior. For example, a few recent studies have correlated the lipid oxidation of an emulsion with respect to the physical and chemical structure of the emulsion’s interface [38]. More information about the physical and chemical barrier effect of interfaces according to their molecular composition and physical organization is needed, and adequate models of oil–water interfaces and their tools for characterization must be developed. The potential to characterize an emulsion’s complex morphological structures including the interface using novel ^1^H LF NMR and reconstruction algorithms is described in subsequent sections.

### 1.4. Comparison of ^1^H LF NMR with Common Methods of Characterizing Oil Oxidation

Numerous chemical and physical analytical methods have been developed to assess lipid oxidation such as peroxide values (PV) and *p*-anisidine (PAV), high performance liquid chromatography (HPLC), gas chromatography–mass spectrometry (GC–MS), Fourier transformation infrared spectroscopy (FTIR), electron spin resonance (ESR) and HR NMR [17,35,39]. There are however deficiencies and lack of consistency in many of the results from these studies. The analytical methods cannot efficiently resolve morphologies and multiple chemical components or they are designed to detect one type of oxidation product while lipid oxidation is complex and generates multiple products at different stages of oxidation. The common methodologies peroxide value (PV) and *p*-anisidine value (PAV) have many drawbacks, such as strict time regimes during individual stages of analyzes, control of reaction conditions and components including light and atmospheric oxygen exposure and large amount of environmental harmful solvents [40]. Though these tests allow the evaluation of either primary or secondary oxidation products anyone tests does not give a measure of both products. Despite the simplicity of the thiobarbituric acid (*TBA*) spectrophotometric test for measuring secondary oxidation products it has many limitations in identifying them [37]. TBA reacts with malondialdehyde (MDA), which is one of the several low-molecular weight end products formed from the decomposition of some primary and secondary lipid peroxidation products. However, not all peroxidation reactions generate MDA, therefore TBA data may be misleading. GC–MS is another common methodology used in the assessment of PUFA’s degree of oxidation, that requires a complex sample preparation characterized by lipid extraction and subsequent derivation steps, which are very time consuming [37].These and other spectroscopic methods such as HF ^1^H NMR and FTIR are limited in the resolution they can achieve of the different chemical and morphological states of PUFA oils and emulsions [7,41]. Hwang [7] suggested the development of methods that combine the concomitant detection of multiple oxidation products for the consistent assessment of lipid oxidation. In this respect, ^1^H LF NMR spectroscopy technology of the present review paper demonstrates a significant potential in readily elucidating the molecular structures and their physical arrangements within different lipid oxidation products.

### 1.5. Applications of ^1^H LF NMR Relaxation in Food Industry

^1^H LF NMR is a rapid non-invasive and non-destructive technology extensively used in the agro, food, polymer, petroleum and pharmaceutical industries [8,34,42]. An important application is in industrial quality control for the determination of solid-to-liquid and oil-to-water ratios in materials, food emulsions and plant seeds [43]. In biological samples, ^1^H spins exist in a variety of different environments, giving rise to a distribution of relaxation times in which the measured relaxation decay is a sum of contributions from all spins [14]. Examples of different applications are measurements of fat content in meat [44], determination of the water content of meat, fish [45] and in agro food products [46], molecular mobility in wheat starch [47], study of protein denaturation in eggs and whey [48], the effect of the formulation on liquid and solid fat ice cream [49] and quality control of food such as avocados [50]. There are also multiple applications in many different food products and peak assignment for exploratory purposes in other foodstuffs including eggs, fish, dairy products, salad cream and cake [51]. As described in subsequent sections these graphs can efficiently monitor oxidation process, potential oxidation stability, and assess antioxidants efficacy in oils and emulsions [24,27,34]. In agreement with Berton-Carabin et al. [38] oils and O/W emulsion’s chemical and morphological arrangements has not been extensively studied and this is one of the main issues that is focused upon in the present review using ^1^H LF NMR and reconstruction algorithms.

## 2. Demonstration of TD Sensor Fingerprint Mapping of FAs, Oils, Seeds and Paste Products

### 2.1. TD Fingerprint Mapping of FAs, Oils, Seeds and Paste Products

**Figure 7:**
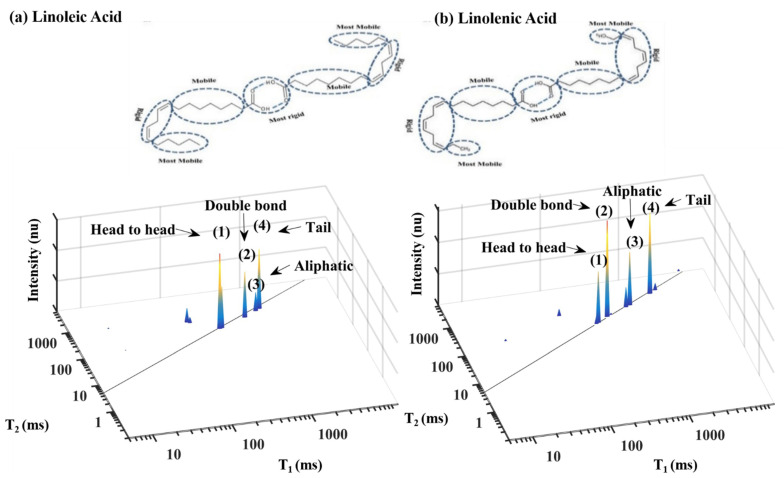
Dimer structure and segmental rigidity-mobility characterization of PUFA. (**a**) Linoleic acid (18:2) and (**b**) linolenic acid (18:3). ^1^H LF NMR TD sensor 2D T_1_-T_2_ graphic mapping and peak assignment is presented in the lower Table 1 for each of the two PUFAs. Reprinted with permission from ref. [27]. Copyright 2019 Wiley.

This section demonstrates TD fingerprint mapping of various basic lipid components in food products. In addition to the FAs dimer head-to-head microstructural morphology, self-assembly as described above in the introduction section (Figure 1 [25]), Figure 7 shows the 2D T_1_-T_2_ LF NMR sensor analysis of two standard PUFA dimers, linoleic acid (18:2) and linolenic acid (18:3). The rigidity/mobility of the different segments in these two PUFAs is marked (Figure 7a,b, respectively). Most rigid head-to-head segments correspond to peak 1 in the T_1_ vs. T_2_ spectra. Second rigid double bonds segment, relatively mobile aliphatic chain segment and most mobile tail domain segments correspond to peaks 2, 3 and 4, respectively, as shown and comprehensively described in the publications by Resende et al. [24,27].

The PUFAs linoleic and linolenic acid have similar spectra with four different peaks and each one of these peaks is correlated with one of the PUFA’s segmental motion TD [21,44], with the major difference being in the mobile terminal aliphatic chains T_1_ and T_2_ values. As tabulated in Table 1, peak 1 had the most rigid/lowest values of T_1_ and T_2_ (T_1_ (18:2) 320 ms and T_2_ (18:2) 292 ms; T_1_ (18:3) 309 ms and T_2_ (18:3) 302 ms), was assigned to the most rigid segment of head-to-head configuration. The second most rigid PUFA segmental motion TD are the double bonds (peak 2 (T_1_ (18:2) 845 ms and T_2_ (18:2) 512 ms; T_1_ (18:3) 442 ms and T_2_ (18:3) 444 ms)). The more mobile aliphatic chain was assigned as the peak 3 (T_1_ (18:2) 1111 ms and T_2_ (18:2) 852 ms; T_1_ (18:3) 1099 ms and T_2_ (18:3) 927 ms). The most mobile PUFA segmental motion TD is the tail, with the longest relaxation times (peak 4 (T_1_ (18:2) 1267 ms and T2 (18:2) 950 ms; T_1_ (18:3) 2288 ms and T_2_ (18:3) 1948 ms)).

**Figure 8:**
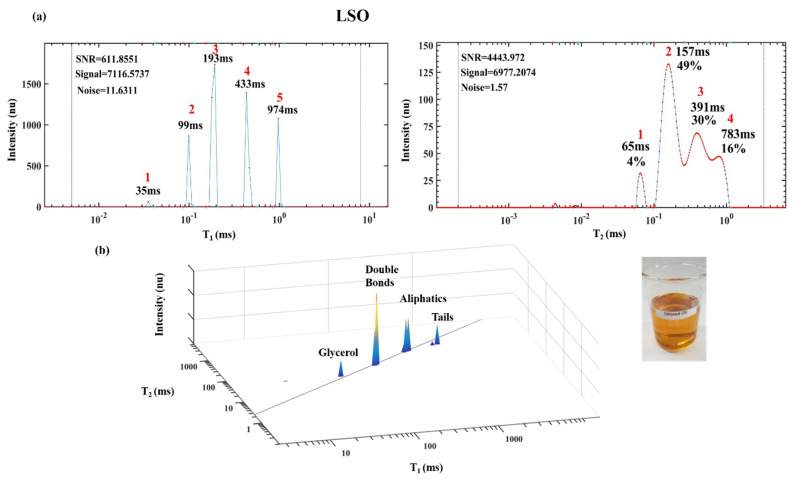
One-dimensional T_1_ and T_2_ spectra of linseed oil (LSO) (**a**) and 2D T_1_-T_2_ spectra (**b**). Image of linseed oil (bottom right). Reprinted with permission from ref. [27]. Copyright 2019 Wiley.

Food oils based on triacylglycerides (TAGs) were also readily characterized by ^1^H LF NMR and T_1_ and T_2_ reconstruction into TD graphics. Figure 8 shows linolenic rich linseed oil’s (LSO) 1D T_1_ and T_2_ and 2D T_1_ vs. T_2_ relaxation time spectra. LSO’s chemical structure is characterized by four different segmental motion TD: glycerol, double bonds, aliphatic chain and tail as shown for the fatty acids in Figure 8 and Table 2. The NMR relaxation analyses gives a spectrum (Figure 8) with four peaks and these peaks were correlated with the PUFA’s rich LSO segmental motions TDs. Glycerol is the most rigid TD and therefore it corresponds to peak 1 with the lowest values of T_1_ and T_2_. Double bonds are the second most rigid TD and assigned to peak 2. The more mobile aliphatic chain is assigned to peak 3 with higher values of T_1_ and T_2_. The tail is the most mobile TD and it is assigned to peak 4 with the highest values of T_1_ and T_2_. These values differ significantly from the T_1_ and T_2_ values of the FAs linoleic and linolenic acids shown in Table 1, because free FAs without a glycerol connectivity, have different self-assembled aggregate structures as would be expected from the TAG’s molecular structures. This demonstrates the efficiency of the chemical and morphological TD fingerprinting technology to characterize different fats and oils.

**Figure 9:**
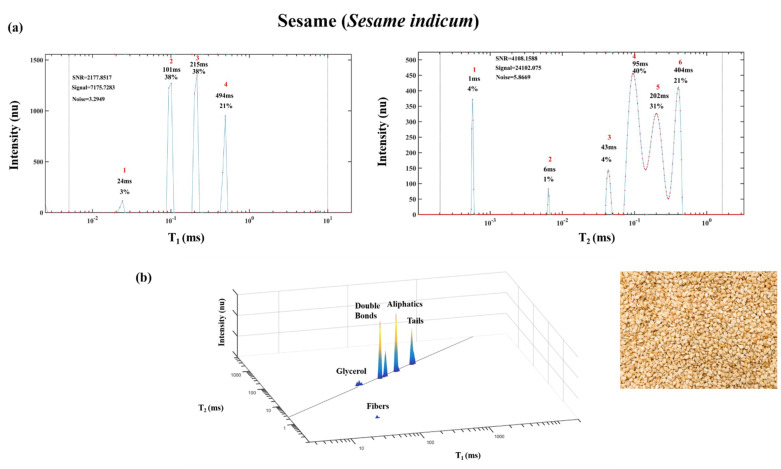
One-dimensional T_1_ and T_2_ spectra of sesame seeds (*Sesamum indicum*) (**a**) and 2D T_1_-T_2_ spectra (**b**). Image of sesame seeds (bottom right).

Figure 9 shows sesame seeds’ oils and fibers 1D T_1_ and T_2_ and 2D T_1_ vs. T_2_ energy relaxation time spectra. The chemical structure of sesame seed oil is characterized by the four lipids different segmental motion TD: glycerol, double bonds and aliphatic chain described above. In this example, an additional segment appears below the diagonal line at lower T_1_ and T_2_ that represents solid fibers as determined based on standards [8]. The large difference between linseed oil (Figure 8) and the T_1_ and T_2_ values of sesame seed oils (Figure 9), shows the potential of characterizing the differences between the oils.

**Figure 10:**
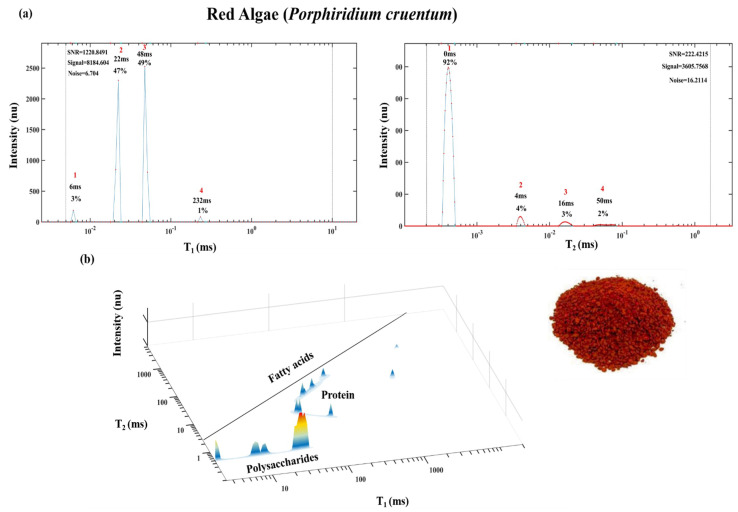
One-dimensional T_1_ and T_2_ spectra of dry *Porphyridium cruentum* sp. (red microalgae) (**a**) and 2D T_1_-T_2_ spectra (**b**). Image of dry red microalgae (bottom right).

**Figure 11:**
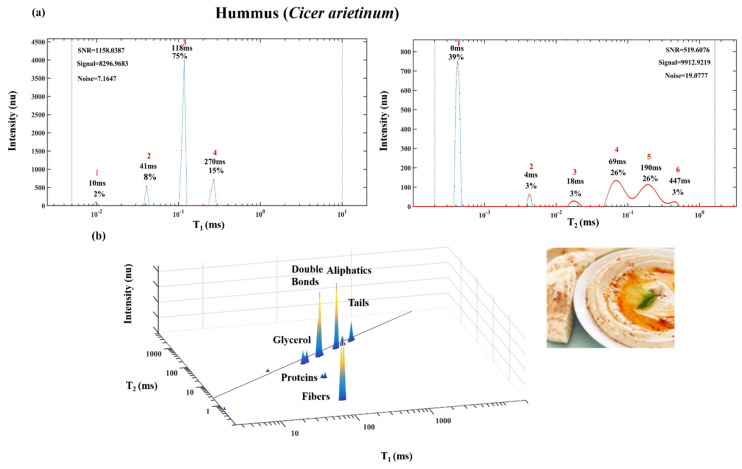
1D T_1_ and T_2_ spectra of *Cicer arietinum* seeds paste (humus) (**a**) and 2D T_1_-T_2_ spectra (**b**). Image of humus paste (bottom right).

As shown below, T_1_ vs. T_2_ spectra that can also be generated for biological aggregate structures such as microalgae (Figure 10), humus seed paste (Figure 11) and many other organic food products.

Figure 10 shows dry red microalgae prepared from *Porphyridium cruentum* sp., 1D T_1_ and T_2_ and 2D T_1_ vs. T_2_ energy relaxation time spectra. The chemical structure of dry microalgae is also characterized by a lipid TD close to the diagonal line, in the 2D spectra. Additional TDs below the diagonal are obtained and assigned to a protein (based on a standard). In the lowest T_2_ level, three TDs according to the increasing levels of T_1_ are obtained. This last group of TDs are assigned as sulfonated polysaccharides most abundant in *Porphyridium cruentum* sp. red microalgae [52]. The lowest T_1_ TD is assigned to the most crystalized polysaccharide and the longest T_1_ is the most amorphous sulfonated polysaccharide [8]. 

Figure 11 shows humus paste product prepared from *Cicer arietinum* seeds with added olive oil, and their 1D T_1_ and T_2_ and 2D T_1_-T_2_ energy relaxation time spectra. The chemical structure of humus product is also characterized by the lipid’s four different segmental motion TD: glycerol, double bonds, aliphatic chain and aliphatic tail as described above. In this paste product, an additional two segments appear below the diagonal line. One segment is assigned, based on the standard, to a protein and the second is assigned to lignocellulose solid fibers.

### 2.2. Demonstration of TD Sensor Fingerprint Mapping of FA Oil Oxidation

The analysis of molecular configuration and aggregate structures of PUFA oils or within their O/W emulsions with respect to its susceptibility and degree of oxidation is important for foods and many other applications because of PUFA oil’s facile oxidation into toxic side products. The oxidation process generates both low molecular weight and volatile side products and polymeric structures. Though ^1^H LF NMR was providing only limited and basic chemical information, it has recently proven to be useful for characterizing dynamical properties of polymer chains [52]. The magnitude of the energy relaxation times T_1_ and T_2_ are dependent upon the interaction between proton spins and their surrounding environment (lattice) that is modulated by molecular mobility. In fact, there is a well-known dependence with the motional correlation time τ_c_, described by the BPP equations (Bloembergen–Purcell–Pound) [53]. Thus, practically for the liquid phase of small molecules including fatty acids and oils, T_1_ and T_2_ values for a given TD are similar. For polymers, proteins and large molecules while T_2_ is continuously decreasing T_1_ is increasing. Taking advantage of this phenomena related to chemical and structural changes, it was possible to develop the present ^1^H LF NMR TD sensor application to monitor a lipid’s oxidation process.

**Figure 12:**
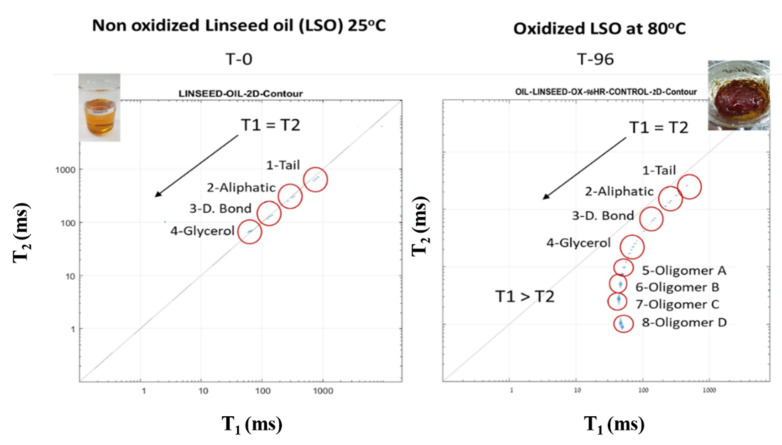
Two-dimensional T_1_-T_2_ fingerprint mapping of LSO before (T-0) and after 96 h of heating at 80 °C. Reprinted with permission from ref. [27]. Copyright 2019 Wiley.

Linseed oil (LSO) oxidation results in structural and physical changes of the oil, which could be followed by our ^1^H LF NMR TD sensor with high efficacy (Resende et al., [10,17]). As LSO is a relatively small molecule the T_1_ and T_2_ have similar values on the diagonal (Figure 12). The glycerol ^1^H is the most rigid TD and therefore is characterized by lower T_1_ and T_2_ values, followed by the double bond and the aliphatic chain TD respectively. The tail is the most mobile TD and therefore corresponds to the peak of highest values of T_1_ and T_2_. After 96 h of oxidation at 80 °C, the 2D T_1_-T_2_ energy relaxation time spectra shows formation of new peaks that are the oxidation products resulting from crosslinking and polymerization in the termination phase of oxidation. The relatively high molecular weight of the oxidation products is characterized by different values of T_1_ and T_2_ per peak, forming the characteristic oxidized bending effect from the T_1_ = T_2_ diagonal in the spectra curve. The four characteristics LSO TDs (peaks 1–4) remain but they have lower values because of the secondary interactions between the non-oxidized LSO and its oxidation products.

**Figure 13:**
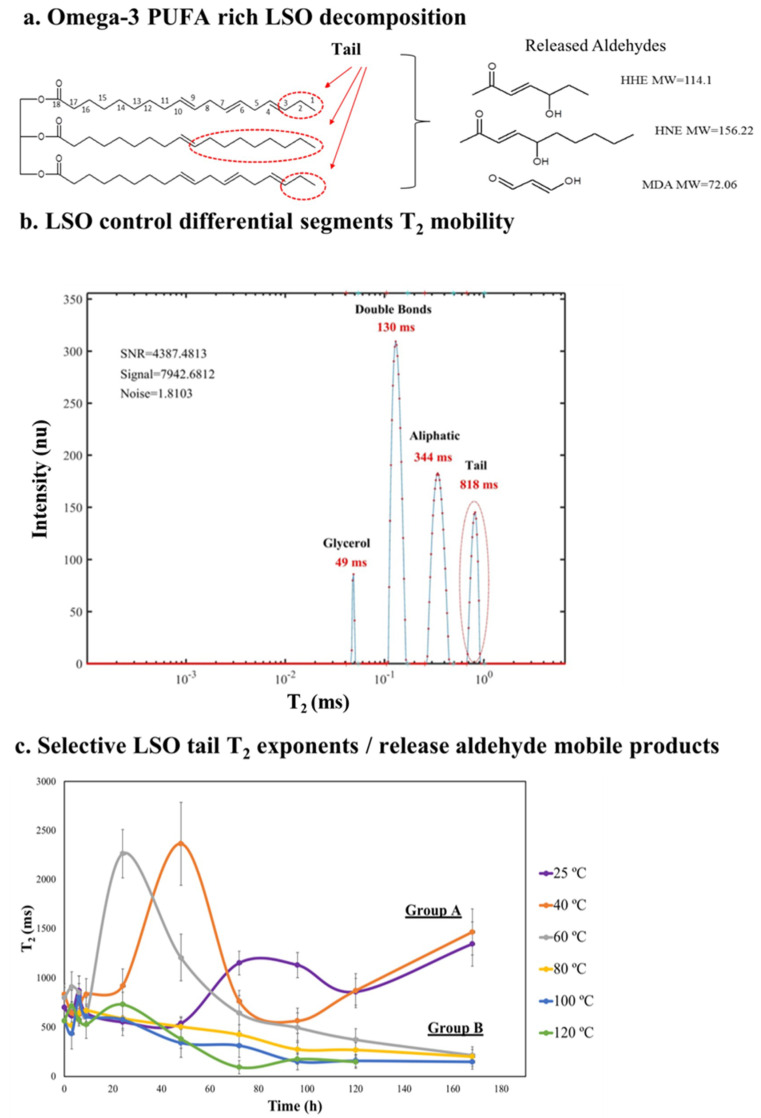
(**a**) Omega-3 linolenic acid-rich LSO decomposition pattern and main released aldehydes as determined by GC–MS analysis, (**b**) selective exponents of LSO segments with emphasis of segment of tail T_2_ time domain relaxation determination (**c**) and graphic presentation of LSO tail T_2_ changes during heating at 25, 40, 60, 80, 100 and 120 °C together with air pumping for 168 h (25 and 40 °C designated as Group A and 60, 80, 100 and 120 °C designated as Group B). Mean ± SD is presented for each T_2_ time point. Reprinted with permission from ref. [24]. Copyright 2019 Wiley.

The speed with which data is obtained and the complexity of the signal acquired can become overwhelming unless suitable methods for interpretation are used. For industrial on-line monitoring of food production with PUFA components, rapid analysis of the oxidation status of the components would be important. In one of our recent studies [34], the T_2_ values of the oil’s aliphatic chain tail oxidation could be rapidly monitored to measure the samples degree of oxidation. The omega-3 linolenic acid-rich LSO molecular structure and the released aldehydes as determined by GC–MS analysis are shown in Figure 13a. The specific T_2_ NMR values of the different LSO molecular components are shown in Figure 13b wherein the tail segment’s T_2_ is used to follow oxidant degradation as shown by the changing T_2_ values in Figure 13c. This is a graphic presentation of LSO tail T_2_ values changes during heating at 25, 40, 60, 80, 100 and 120 °C, together with air pumping for 168 h (25 and 40 °C is designated as Group A and 60, 80, 100 and 120 °C designated as Group B). 

Figure 13a shows a simplified scheme of LSO oxidation decomposition. LSO is composed mainly of the FA linolenic acid (55%). The LSO chemical structure is a triacylglycerol (TAG) with two linolenic acid and one oleic acid. The FA’s tails are circled in red, that during oxidation cleave forming the volatile low molecular weight aldehyde malonaldehyde (MDA) and the high molecular weight non-volatile aldehydes 4-hydroxy-trans-2-nonenal (HNE) and 4-hydroxy-trans-2-hexanal (HHE) that remain within the oxidized oil and react with other oxidation products forming highly cross-linked polymer products that increase the oxidized oils viscosity.

Figure 13b presents a 1D T_2_ relaxation time spectra of LSO. Although the 2D T_1_-T_2_ spectra was reported to efficiently characterize PUFA rich material’s oxidation [24,27], its relatively long acquisition time (e.g., T_2_ 20 s vs. 20 min for T_1_) limits its application for on-line and at-line industrial production processes [7]. Considering that T_1_ and T_2_ have almost equal values for relatively small molecules such as TGA, but T_1_ has technically a longer acquisition time, the 1D T_2_ TD is more appropriated for a rapid NMR relaxation methodology for monitoring of LSO oxidation. Data processing based on PDCO is able to efficiently reconstruct the T_2_ NMR signal onto spectra. The T_2_ reconstructed spectra characterizes both chemically and morphologically the different segmental sections of LSO: glycerol, double bonds, aliphatic chain and tail. Glycerol is the most rigid segment, and it is assigned to peak 1 with the shortest T_2_ relaxation time. Double bonds are the second most rigid segments and they assigned to peak 2. Aliphatic chains are more mobile and corresponds to peak 3. Highlighted in red the most mobile segment is the aliphatic tail chain that corresponds to peak 4 with the longest T_2_.

This study suggests a methodology that uses TD tail T_2_ values as a marker for a rapid assessment of PUFA’s rich oil oxidation such as LSO. Figure 13c shows LSO tail T_2_ values during oxidation at 25, 40, 60, 80 and 120 °C during 168 h. The LSO tail segments T_2_ before oxidation was approximately 750 ms. The oxidation curves were separated into two groups: A (slow oxidation) and B (rapid oxidation).

**Figure 14:**
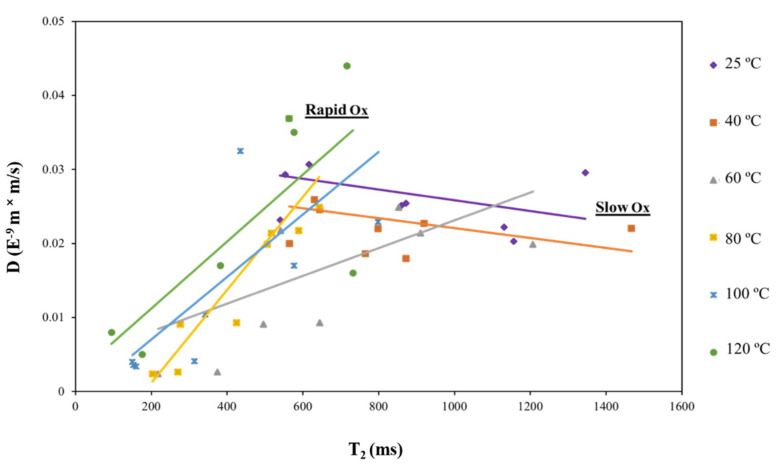
Correlation between LSO self-diffusion (D) and tail T_2_ at 25, 40, 60, 80, 100 and 120 °C during 168 h. The points of each applied temperature (different color) represent the distribution of D-T_2_ values in each time tested along the 168 h and the best straight line for each oxidation temperature is drawn. (25 and 40 °C designated as Slow Ox, and 60, 80, 100 and 120 °C designated as Rapid Ox). Reprinted with permission from ref. [34]. Copyright 2019 Wiley.

To further support this last finding, self-diffusion tests using ^1^H LF NMR were carried out for each of the samples. Self-diffusion provides good and accurate physical information regarding the movement and mixing capability of the components included in the sample [16,34]. It is well known to correlate with the samples measured viscosity (not shown). Figure 14 shows the correlation between the NMR self-diffusion (D) values and the T_2_ tail values of the LSO oxidized at 25, 40, 60, 80, 100 and 120 °C during 168 h. The results show that the LSO autoxidation follows two different patterns according to the oxidation temperature. The LSO samples oxidized at low temperatures (25 and 40 °C) have a T_2_ vs. D curve with a negative slope, which can be rationalized by the slow LSO oxidation rate, and formation of low MW products, which decrease viscosity. LSO samples, however oxidized at higher temperatures (60 80, 100 and 120 °C) are characterized by a positive T_2_ vs. D slope, which can be explained by the rapid LSO oxidation rate and formation of high MW products that increase viscosity. The temperature of 60 °C although also presents a positive slope, it is more moderated than the ones of higher temperatures. Therefore, 60 °C is considered the initial point for a rapid LSO oxidation [34]. 

### 2.3. Demonstration of TD Sensor Fingerprint Mapping of Food O/W Emulsion Products and Oxidation

Linseed O/W emulsions (LSEs) of the present study are made from linseed oleosomes (oil bodies, OB) as described in detail by Resende et al. [41]. In Section 2.2 the structural organization of LSE vesicles was described as oil encapsulated on the inside by different interfacial components such as polar head groups of amphiphiles, with their hydrophobic tails within the vesicle’s core isolated from the continuous aqueous matrix environment. The interfacial surface of LSE’s vesicles has phospholipids, residual linseed mucilage components and amphiphilic proteins such as oleosins, caleosins and steroleosins [54,55]. These interfacial surface components are responsible for the physical stability of the OB’s vesicles and therefore the emulsion’s stability. In the present review paper, the chemical and morphological structures of the linseed emulsions (LSE) and LSE with coencapsulated fish oil (LSFE) are characterized in terms of the OB’s vesicles chemical and morphological structures with a multicomponent amphiphilic surface and lipophilic core of encapsulated PUFA-rich oil. Linseed OB aggregates have structural components that are postulated to enhance the stability against oxidation of the internal encapsulated PUFA oils [38].

**Figure 15:**
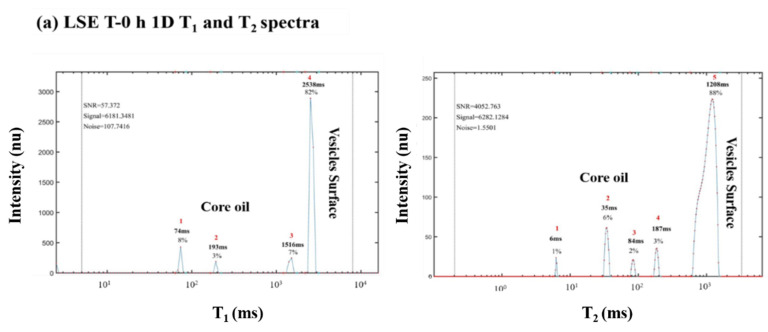

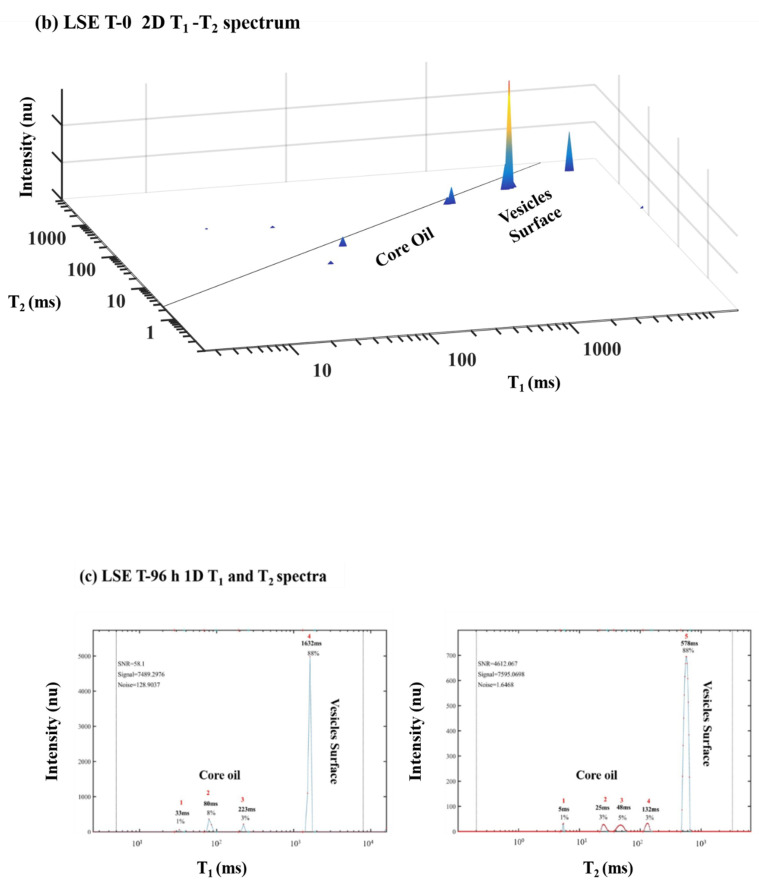

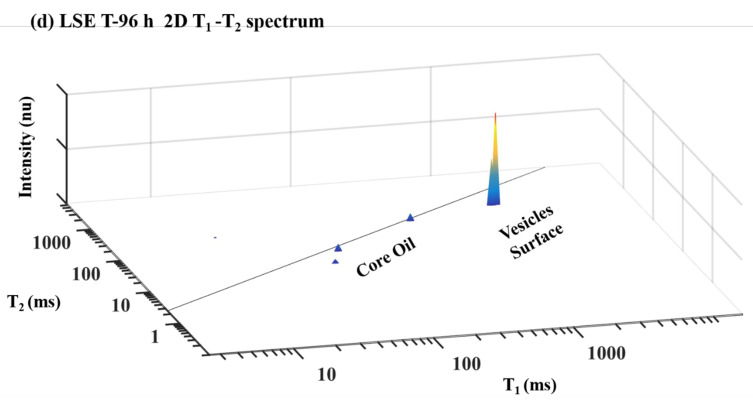
^1^H LF NMR relaxation spectra of LSE fresh produced (T-0 h **a**,**b**) and after 96 h of thermal oxidation at 55 °C (T-96 h, **c**,**d**). Two-dimensional T_1_-T_2_ peaks values and proposed ^1^H TD assignment are shown in Table 3.

^1^H LF NMR relaxation was used to characterize chemical and structural LSE O/W emulsions before and after thermal oxidation. Figure 15 shows the 1D T_1_ and T_2_ and 2D T_1_-T_2_ relaxation time spectra and Table 3 numerical TD values of LSE before and after 96 h of thermal oxidation at 55 °C. ^1^H LF NMR relaxation sensor analysis indicates in the T_1_-T_2_ fingerprinting map two main TDs: one is the TAG molecules inside the vesicle’s core and the other is the vesicle’s interfacial surface. The 1D T_1_ relaxation time spectra for LSE T-0 h and LSE T-96 h show three small peaks that are postulated to correspond to the TAG molecules inside the OB’s core and one peak of higher intensity that corresponds to the OB’s interfacial surface. The high peak of the surface is explained by the fact that the outer surface layer of the OB particles contains a significantly higher level of protons in comparison to the OB’s core encapsulated hydrophobic oil, due to the bound and associated water. The 1D T_2_ relaxation time spectra for LSE T-0 h and LSE 96 h present four small peaks that are assigned as the PUFA-rich oil molecules within the OB’s core and one peak of higher intensity that corresponds to the OB’s interfacial surface. The 2D T_1_-T_2_ relaxation time spectra of LSE T-0 h and LSE T-96 h show three small peaks assigned as the PUFA-rich oil molecules within the OB’s core and one peak of higher intensity that corresponds to the OB’s interfacial surface. It is interesting to note that the emulsion LSE before and after 96 h of oxidation have similar relaxation patterns. However, the T_1_ and T_2_ peaks values presented in the table in Figure 15, showed that the sample of LSE after T 96 h oxidation has somewhat lower relaxation times than the fresh LSE sample at T 0 h. This is most probably due to some thermal oxidation and resulting increase in internal viscosity. The degree of oxidation, however within the LSE emulsions extracted from the oil body has significantly less oxidations according to the changes in T values before and after oxidation, than LSO shown in Figure 12 and Figure 14.

Using other conventional analyses of physical and structural properties of non-heated (T-0) and heated (T-96) LSE well supports the explanation of results obtained by ^1^H LF NMR TD relaxation sensor. Testing of self-diffusion of LSE samples before and after thermal heating for 96 h shows a small decrease from 2.750 to 2.383 10^−9^ m*m/s, respectively, suggesting a small viscosity increase of the LSE emulsion. Dynamic light scattering (DLS) analysis shows an increase of OB size from 803.2 nm at T 0 h to 1363 nm after T 96 h of thermal heating. This increase in particle size can be correlated to the decrease in peak intensity for the major peak in the oxidized sample vs. that of the major peak in the non-oxidized initial sample, as these peaks are due to the interfacial ^1^H and thus total emulsion particle size. Furthermore, the results of zeta potential assay show values of −29.1 mV and −28.7 mV for T 0 h and T 96 h, respectively. Usually, it is accepted that emulsion preparations with values of zeta potential above −40 mV are considered as very stable and values of about −30 mV are considered to represent moderate stability [56].

### 2.4. Demonstration of TD Sensor Fingerprint Mapping of Oleosome Oil Bodies Encapsulating of External Oils

There is increasing interest in using oil bodies as natural emulsions and novel carriers for the delivery of lipophilic bioactive molecules [56]. During the preparation of linseed oil body emulsions, and oil bodies of other seeds, the addition of external oils such as fish oil (FO) with omega-3 FA EPA and DHA, can be coencapsulated and coprotected with linseed oils against oxidation. In the present review paper, the chemical and morphological structures of the linseed emulsions (LSE) with coencapsulation of fish oil (LSFE) are described and characterized in terms of the OB’s vesicles chemical and morphological structures with a multicomponent amphiphilic surface and lipophilic core of PUFA-rich coencapsulated fish oil.

**Figure 16:**
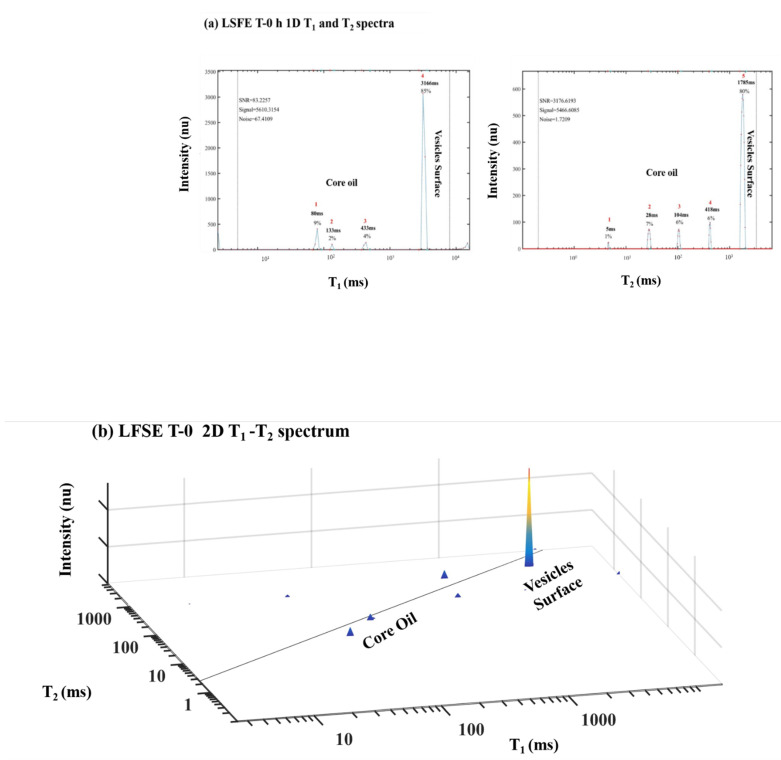

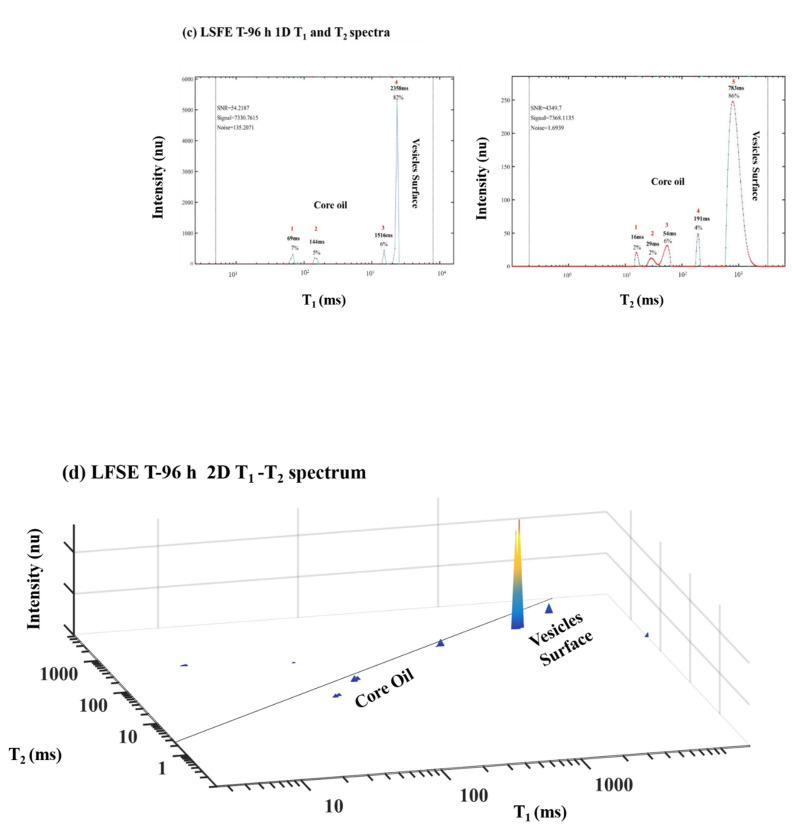
^1^H LF-NMR relaxation spectra of LSFE fresh produced (T-0 h, **a**,**b**) and after 96 h of thermal oxidation at 55 °C (T-96 h, **c**,**d**).

LSFE was prepared by the addition of FO, rich with long chain PUFAs, EPA and DHA. The chemical and morphological structure of LSFE is characterized by OB vesicles of linseed, with an amphiphilic interface surface and lipophilic core encapsulating PUFA-rich LSO and FO. The ^1^H LF NMR energy relaxation TD fingerprinting for LSFE before (T 0 h) and after thermal oxidation at 55 °C for 96 h (T 96 h) are shown in Figure 16.

It is interesting, that before and after thermal oxidation at 55 °C for 96 h only minimal phase separation could be visually observed in the LSFE samples. ^1^H LF NMR TD sensor analysis as seen in Figure 16 shows that LSFE T 0 h and LSFE T 96 h had similar T_1_ and T_2_ TD graphic pattern, as was obtained for LSE in Figure 15. The 2D T_1_-T_2_ relaxation TD fingerprint maps of LSFE T 0 h and LSFE T 96 h show small peaks assigned as the PUFA-rich oil molecules within the OB core and one peak of higher intensity related to the energy relaxation time on the OB’s vesicle surface.

The Table in Figure 16 of the T_1_ and T_2_ numerical TD values of LSFE T 0 h and LSFE T 96 h indicates that the values of T_1_ and T_2_ of the main peak assigned to the vesicle’s surface were somewhat lower for LSFE after the thermal oxidation of 96 h as were most of the other peaks that could be observed. This data had the same general pattern obtained for LSE (Table 4).

**Figure 17:**
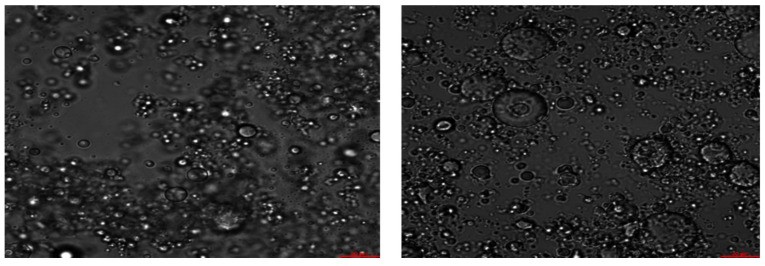
Confocal microscopic images of LSFE OB emulsion of non-heated sample (**left** T-0, X63) and after 96 h of thermal heating (55 °C) (**right** T-96. X63).

Microscopic images of LSFE OB emulsion before and after 96 h of thermal heating (Figure 17) shows some small increase of OB size upon heating. In both cases before and after heating only minimal phase separation could be observed in the LSFE samples. These results were similar to the microscopic images shown for LSE.

Similarly, to the pattern obtained for LSE without FO, the sample of LSFE shows some increase of droplet particle size distribution from 1374 to 1951 nm for fresh and heated sample (Table 5) respectively. Zeta potential values for LSFE were −27.3 and −25.1 mV for the two samples, suggesting a moderate stability of the emulsion samples. The rate of self-diffusion was slightly reduced from 2.902 to 2.734 10^−9^ m*m/s, respectively, suggesting again a moderate increase of vesicle size and/or internal viscosity. Furthermore, results of PV tests correlate well with all the previous data regarding LSE and LSFE samples, and only a small increase of PV strongly supports to the efficacy of a rapid non-destructive ^1^H LF NMR TD sensor application to monitor oxidation in complex materials as demonstrated in the present study. The relatively small increase in oxidation products within the samples can be seen by the T_1_/T_2_ ratio from 2.07 to 2.61. In addition, considering the reports of emulsion preparation and stability at low refrigerated conditions (4–7 °C) [57], the natural linseed OB emulsion encapsulation PUFA-rich components including FO appears to be relatively stable with high oxidant resistance even under relatively high thermal oxidizing conditions. 

## 3. Summary and Conclusions

In addition to individual PUFA molecules having specific molecular structures, their material functionality and their response to oxidants are strongly dependent on their aggregate structures in food oils or within aqueous emulsions. As shown the chemical and morphological structures of complex aggregate materials can be efficiently monitored by ^1^H LF NMR and used to measure oxidation and assess antioxidants efficacy. In this review paper we describe the efficacy of the ^1^H LF NMR and data reconstruction programs in analyzing the chemical and morphological structure of PUFA-rich oils and O/W emulsions of PUFA-rich oleosome-oil bodies from seeds, with and without encapsulation of externally added oils, and their susceptibility to oxidant degradation as a function of their chemical and morphological structure. We demonstrate a facile ^1^H LF NMR energy relaxation time domain (TD) sensor technology for direct non-modified sample analysis. A prime dual convex objectives (PDCO) optimized solver was used for computational processing of ^1^H LF NMR energy relaxation curves into the energy relaxation time signals of spin-lattice T_1_ and spin-spin T_2_, for 1D or a 2D graphical fingerprinting maps of oils or oil in water emulsions. The individual T_1_ or T_2_ or T_1_ vs. T_2_ peaks correlated to structural or morphological domains within the material. With this material analysis the molecular configuration and aggregate structures of unsaturated FA oils or FAs within oil/water emulsions can be readily characterized with respect to their chemical and morphological domains, which influences oxidation, and can assess antioxidants efficacy of the components and system. For industrial on-line monitoring of food production with PUFA components, rapid analysis of the components’ oxidation status would be important. It was shown that the T_2_ values of the oil’s aliphatic chain tail oxidation could be rapidly monitored to measure the samples degree of thermal oxidation. LSO samples oxidized at low temperatures (25 and 40 °C) have a T_2_ vs. D curve with a negative slope, which can be rationalized by the slow LSO oxidation rate, and formation of low MW products, which decrease viscosity. LSO samples oxidized at higher temperatures (60 80, 100 and 120 °C) are characterized by a positive T_2_ vs. D slope, which can be explained by the rapid LSO oxidation rate and high MW products that increase viscosity. The temperature of 60 °C although also presents a positive slope, it was more moderated than the ones of higher temperatures. Therefore, 60 °C was considered the initial point for a rapid LSO oxidation.

^1^H LF NMR analysis of O/W emulsions of PUFA-rich oleosome-oil bodies from seeds show only minimal chemical and structural changes. This well explains linseed emulsion (LSE) without and with fish oil (LSFO) samples significant oxidative stability. These findings were supported by other conventional microscopic and spectral technologies.

The ^1^H LF NMR TD technology sensor proved to be an effective tool to characterize and monitor PUFA oxidation, and to potentially identify optimal formulations and preparative methods of PUFA aggregate structures. We also demonstrated the potential of characterizing food products such as seeds (sesame seeds) and paste products (e.g., humus paste) and red microalgae not only with respect to the oil contents but also their polysaccharides and protein content. Future direction of this work is to advance the application of this ^1^H LF NMR sensor for quality control with emphasis of monitoring oxidation process of food products.

## Figures and Tables

**Table 1:** ^1^H LF NMR T_1_-T_2_ TD numerical values of linoleic (18:2) and linolenic acids (18:3).

Peak	Linoleic Acid		Linolenic Acid	
	T_1_ (ms)	T_2_ (ms)	T_1_ (ms)	T_2_ (ms)
1	320 ± 10	292 ± 10	309 ± 29	302 ± 33
2	845 ± 35	512 ± 29	442 ± 54	444 ± 35
3	1111 ± 59	852 ± 34	1099 ± 207	927 ± 103
4	1267 ± 68	950 ± 22	2288 ± 192	1948 ± 113

**Table 2:** Numerical values of T_1_-T_2_ of LSO and peak assignment.

Peak	T_1_ (ms)	T_2_ (ms)	Dictionary
1	94	53	Glycerol
2	191	135	Double Bonds
3	437	344	Aliphatic Chain
4	1003	766	Tail

**Table 3:** Numerical values of T_1_-T_2_ of LSE and proposed ^1^H TD assignment.

Peak	T-0		T-96		^1^H TD Assignment
	T_1_ (ms)	T_2_ (ms)	T_1_ (ms)	T_2_ (ms)	
1	2941	1287	-	-	Surface (small OB)
2	2732	1206	1756	615	Surface (medium OB)
3	905	577	374	307	FA-Aliphatic chains
4	92	55	80	57	FA-D. Bonds
5	59	18	64	25	Glycerol

**Table 4:** Two-dimensional T_1_-T_2_ peaks values of LSFE and proposed ^1^H TD assignment.

Peak	T-0 h		T-96 h		^1^H TD Assignment
	T_1_ (ms)	T_2_ (ms)	T_1_ (ms)	T_2_ (ms)	
1	64	29	55	21	Glycerol
2	115	84	92	57	FA D. Bonds
3	726	282	583	416	FA Aliphatic chains
4	3948	1902	2538	971	OB surface

**Table 5:** Physical–structural properties of non-heated and heated LSFE+SD.

	T-0	T-96
T_1_/T_2_	2.07 *	2.61 *
Self-diffusion (10^−9^ m*m/s)	2.902 ± 0.020	2.7338 ± 0.025
DLS (nm)	1374 ± 81	1951 ± 89
Z Potential (mV)	−27.3 ± 6.6	−25.1 ± 8.3

* SD was not obtained.

## Data Availability

Not applicable.
